# Exploratory evidence for differences in GABAergic regulation of auditory processing in autism spectrum disorder

**DOI:** 10.1038/s41398-023-02619-8

**Published:** 2023-10-18

**Authors:** Qiyun Huang, Hester Velthuis, Andreia C. Pereira, Jumana Ahmad, Samuel F. Cooke, Claire L. Ellis, Francesca M. Ponteduro, Nicolaas A. J. Puts, Mihail Dimitrov, Dafnis Batalle, Nichol M. L. Wong, Lukasz Kowalewski, Glynis Ivin, Eileen Daly, Declan G. M. Murphy, Gráinne M. McAlonan

**Affiliations:** 1 Department of Forensic and Neurodevelopmental Sciences, Institute of Psychiatry, Psychology & Neuroscience, King’s College London, London, UK; 2 Institute for Translational Neurodevelopment, Institute of Psychiatry, Psychology & Neuroscience, King’s College London, London, UK; 3 Research Center for Brain-Computer Interface, Pazhou Lab, Guangzhou, China; 4 Institute for Nuclear Sciences Applied to Health (ICNAS), Coimbra Institute for Biomedical Imaging and Translational Research (CIBIT), University of Coimbra, Coimbra, Portugal; 5 School of Human Sciences, University of Greenwich, London, UK; 6 Department of Basic and Clinical Neuroscience, Institute of Psychiatry, Psychology & Neuroscience, King’s College London, London, UK; 7 MRC Centre for Neurodevelopmental Disorders, King’s College London, London, UK; 8 Department of Psychology, The Education University of Hong Kong, Hong Kong, China; 9 South London and Maudsley NHS Foundation Trust Pharmacy, London, UK

**Keywords:** Autism spectrum disorders, Predictive markers, Predictive markers

## Abstract

Altered reactivity and responses to auditory input are core to the diagnosis of autism spectrum disorder (ASD). Preclinical models implicate ϒ-aminobutyric acid (GABA) in this process. However, the link between GABA and auditory processing in humans (with or without ASD) is largely correlational. As part of a study of potential biosignatures of GABA function in ASD to inform future clinical trials, we evaluated the role of GABA in auditory repetition suppression in 66 adults (*n* = 28 with ASD). Neurophysiological responses (temporal and frequency domains) to repetitive standard tones and novel deviants presented in an oddball paradigm were compared after double-blind, randomized administration of placebo, 15 or 30 mg of arbaclofen (STX209), a GABA type B (GABA_B_) receptor agonist. We first established that temporal mismatch negativity was comparable between participants with ASD and those with typical development (TD). Next, we showed that temporal and spectral responses to repetitive standards were suppressed relative to responses to deviants in the two groups, but suppression was significantly weaker in individuals with ASD at baseline. Arbaclofen reversed weaker suppression of spectral responses in ASD but disrupted suppression in TD. A post hoc analysis showed that arbaclofen-elicited shift in suppression was correlated with autistic symptomatology measured using the Autism Quotient across the entire group, though not in the smaller sample of the ASD and TD group when examined separately. Thus, our results confirm: GABAergic dysfunction contributes to the neurophysiology of auditory sensory processing alterations in ASD, and can be modulated by targeting GABA_B_ activity. These GABA-dependent sensory differences may be upstream of more complex autistic phenotypes.

## Introduction

Altered sensory reactivity, interests, and/or responses in autism spectrum disorder (ASD) are now recognized as core to this diagnosis (Diagnostic and Statistical Manual of Mental Disorders, Fifth Edition) [1]; and are among the earliest indicators for ASD [2, 3]. Auditory features include both hyper- and hypo-responsivity to sounds: for example, excessive and adverse reactions to unexpected loudness [4, 5] and reduced orientation to ‘motherese’ (speech usually directed to infants) [6]. Sensory, including auditory seeking behaviors have also been reported in children with ASD [7]. Behavioral sensory features are therefore assumed to be underpinned by altered processing of sensory signals, however, their neurobiological basis is poorly understood [8–10]. To date, no pharmacological interventions targeting auditory (or any other core) alterations in ASD have been successfully developed.

Nevertheless, there is an accumulation of evidence that ASD is associated with an alteration in excitation–inhibition ratio (E-I imbalance) in the central neural systems [11], especially within the inhibitory ϒ-aminobutyric acid (GABA) pathways [12]. Autistic-like behaviors in genetic models of ASD are reported to be mediated by targeting the GABA system [13–15]. Alterations in bulk tissue GABA concentrations [16], development of GABAergic neurons [17, 18], GABA-dependent brain actions [19], and post-mortem markers [20] have also been supported in individuals with ASD. Thus, the involvement of the GABA system in ASD has had continuing support.

The importance of the GABA system in ASD and related conditions led to the development of arbaclofen, a selective GABA type B (GABA_B_) receptor agonist, originally as a candidate for treatment of the fragile X syndrome, the most common genetic cause of ASD [21]. Subsequent clinical trials of arbaclofen in idiopathic ASD failed to reach the primary outcome, but did show some promise on secondary endpoints, suggesting that future work should use an E/I biomarker to link the subgroup who responded to arbaclofen with the mechanism of action of arbaclofen [22]. In this study, we use arbaclofen as a drug probe of GABA-dependent sensory processing with the hope that this experimental approach can contribute to the evidence base which informs future approaches that may be useful to some autistic individuals.

The brain continually adapts to sensory inputs, filtering out irrelevant stimuli and prioritizing deviant stimuli that may be meaningful (signaling danger or reward) [23]. In the auditory domain, neurophysiological responses to sounds adapt (reduce) to repeated, predictable sounds but increase to novel, unpredictable stimuli [24, 25], a ‘mismatch’ fundamental for auditory perception. Processing of repeated and novel stimuli in the auditory domain can be measured using the oddball paradigm, in which a train of identical repeated sounds (standards) is randomly interrupted by an oddball sound (deviant). The difference between electroencephalogram (EEG) recordings that capture responses to standards and deviants is termed as ‘mismatch negativity’ (MMN) [26–30].

Though the molecular and circuit mechanisms supporting MMN remain uncertain, there is consensus that at least two mechanisms are essential for its generation: 1) Stimulus-specific adaptation (SSA) in animals or repetition suppression in humans allows neural responses to adapt (reduce) to repeated sounds while maintaining responsiveness to deviants [31]. 2) Deviance detection generates increased responses to novel stimuli. MMN has been explained under a predictive coding framework of Bayesian perceptual inference [32, 33] in which the auditory system is hierarchically organized. Each level sends ‘back’ predictions to aid the suppression of ascending neuronal activity evoked by anticipated sounds at downstream levels and sends ‘forward’ a prediction error signal upstream when failure to predict bottom-up information happens. An alternative explanation is that selective feedforward adaptive filtration occurs for synapses that mediate the familiar stimulus but not for those synapses that process the novel stimulus [34, 35]. Under either framework, repetition suppression and deviance detection must co-exist; as verified by decomposing the auditory mismatch response in preclinical animals [36].

In humans, neurodevelopmental conditions such as ASD alter brain function across the subcortical and cortical systems known to be involved in sensory processing from birth [37, 38]. However, oddball results in ASD are heterogeneous. A majority of studies have limited analysis to the event-related response MMN. Smaller amplitudes and/or delayed latencies [39, 40], or no differences [41] in MMN have been reported in individuals with ASD relative to individuals with typical development (TD). Age partially accounts for this variability. Children with ASD tend to have smaller MMN amplitudes relative to TD, whereas adults have comparable measures [42].

Inconsistent results from the oddball paradigm in ASD could also be a consequence of the multi-source distribution of MMN generators and the metric used. Both repetition suppression and deviance detection have been observed in neurons along the hierarchical auditory pathway, including cortical (auditory and medial prefrontal) and subcortical (inferior colliculus and medial geniculate body) regions [43–45]. Work in rodents suggests that the influence of these two components on the MMN is region specific [36]. Repetition suppression is most prominent in the subcortical components and can be pharmacologically modulated in preclinical animals by targeting GABA signaling pathways [46, 47]. In contrast, deviance detection is most prominent in cortical regions [36] and is modulated by N-methyl-D-aspartate receptor activity [48–50]. In humans, the MMN is conventionally quantified by subtracting the standard event-related potential (ERP) from the deviant ERP, and is thought to best reflect cortical-based deviance detection. This conventional event-related measurement therefore provides only a brief snap-shot of the response to auditory stimulation in the time domain. Perhaps unsurprisingly, such limited capture of the components operating during processing of repeated and novel auditory stimuli explains inconsistent results.

In contrast, time-frequency analysis, namely Event-Related Spectral Perturbation (ERSP) [51], assesses changes in underlying neuro-oscillatory dynamics in response to standards and deviants [52–54]. Neuro-oscillatory activities in specific frequency bands have been linked to GABA [55, 56] and provide a foundation for functional connectivity across brain regions supporting cognition and behavior [57]. These observations may be critically relevant for ASD as GABAergic differences are frequently reported in this condition [11] and altered functional connectivity across brain networks is a replicable autistic feature [58]. To the best of our knowledge, no-one has directly tested the hypothesis that alterations in the neuro-oscillatory responses to auditory stimuli are under pinned by differential function of the GABAergic system in ASD.

Here we investigated how altering GABA function modulates auditory processing in individuals with and without ASD. Scalp EEG was used to record neurophysiological responses induced in an auditory oddball paradigm—a stream of regular repeating standard sounds was occasionally interrupted by sounds deviating in frequency, duration, or frequency-duration combination [59]. Participants were tested at placebo (baseline) and after a single oral dose of 15 mg or 30 mg arbaclofen (STX209), a selective GABA_B_ receptor agonist [22, 60]. Although, a conventional ERP analysis compared the MMN in TD and ASD at baseline and during GABA_B_ agonism, we moved beyond to examine the ERSP time-frequency response to repeating standard tones and occasional deviants.

Given the evidence for weaker suppression of repetitive stimuli in ASD [2, 61, 62] and reports of ASD-related alterations in the GABA system [14, 63, 64], we hypothesized that repetition suppression of oscillatory responses to standard tones would be (i) weaker in ASD than TD at baseline and (ii) differentially modulated by challenging the GABA system in ASD and TD.

## Materials and methods

### Study design

This study was implemented as part of a series of case-control experiments to test the hypothesis that there is a difference in GABA pathway function in people with and without ASD [64]. The Medicines Health and Care Regulatory Agency in the U.K. confirmed that this study was “not a Clinical Trial of an Investigational Medicinal Product (IMP) as defined by the EU Directive 2001/20/EC. For transparency, the study was registered on clinicaltrials.gov, which accepts a range of study designs, not only Clinical Trials: https://clinicaltrials.gov/ct2/show/NCT03594552. Participants provided written informed consent approved by King’s College Research Ethics Committee (RESCM-17/18-4081). To examine the acute effects of GABA_B_ modulation, participants (TD and ASD) were given placebo or a single oral dose of 15 or 30 mg of arbaclofen (STX209) on the study day. The compound was donated by Clinical Research Associates (CRA) which is a non-profit subsidiary of the Simons Foundation. CRA holds patents for arbaclofen use in autism (expiring 2027). The oddball task was performed once per visit, three hours after placebo/drug administration within arbaclofen half-life [65]; thus, the task was within the active physiological window. The order of administration of study drug or placebo was randomized to prevent order effects. Visits were at least one week apart to ensure drug wash-out. The present study does not address the use of arbaclofen as a treatment for ASD, but uses the drug to investigate whether there are GABAergic differences in sensory processing in ASD.

Medical cover was provided and participants were asked to remain at our unit at least four hours after drug/placebo intake. The medic was ‘blind’ to the order of administration but had access to the visit placebo/drug allocation information if needed (it was not). If a participant had experienced side effects that were more than moderate in the opinion of the study clinician and after discussion with the Chief Investigator, unblinding occurred to try to avoid exposure to a higher dose of arbaclofen on a subsequent visit.

### Participants

Participants aged 19–53 years old had an intelligence quotient (IQ; Wechsler Abbreviated Scale of Intelligence-II [66]) > 70. Demographics including biological sex and IQ did not differ between TD and ASD groups (Table 1). ASD traits were assessed across both groups using the Autism Quotient (AQ) [67]. We also extracted responses to AQ question 5: “I often notice small sounds when others do not” and coded “strongly disagree; somewhat disagree; somewhat agree; strongly disagree” from 1 to 4, respectively. Please see Supplementary Information for details on inclusion and exclusion criteria.

**Table 1:** Participant demographic data and ASD clinical scores.

Measure	TD	ASD	Statistic	*p*
Number (male/female)	22/16	20/8	*X*^2^ = 1.3	0.26
Age	28.6 ± 8.1	34.8 ± 10.1	*t* = 2.7	0.01
Full-scale IQ	120.2 ± 10.5^a^	117.4 ± 10.4^b^	*t* = 1	0.32
AQ	16.9 ± 8.1^c^	35.1 ± 7.5^d^	*t* = 9.1	7 × 10^–13^
AQ-Q5	2.1 ± 1^c^	3.6 ± 0.6^d^	*t* = 6.4	2.5 × 10^–8^

Values are shown as means ± SD. Group difference of age, IQ, and AQ (autism quotient) scores tested using independent-sample *t* tests; comparison of proportion of males and females tested using Chi-squared test. As a result of Covid lockdown restrictions/participant preference it was not possible to complete in-person IQ testing on ^a^two neurotypicals; ^b^six autistics. In the expert opinion of the team, IQ > 70 based on education/employment. AQ data not returned for ^c^three neurotypicals; ^d^one autistic. AQ-Q5 is score of question 5 of AQ: “I often notice small sounds when others do not”.

### Auditory oddball paradigm

On each visit, a total of 1,400 auditory stimulus trials were presented in an oddball paradigm. The stimulus train comprised sinusoidal tones: 82% standard trials (1000 Hz, 50 ms), 6% frequency deviants (1200 Hz, 50 ms), 6% duration deviants (1000 Hz, 100 ms), and 6% combined frequency-duration deviants (1200 Hz, 100 ms) in random order generated by the random function in MATLAB 9.2.0. The inter-stimulus interval (ISI) was randomized between 500 and 600 ms for each trial of the standard and the deviant tones to prevent anticipation. The regularity imposed by the standards therefore was expected to mainly reflect features of standard tones [39, 54]. Sounds were presented at 70 decibels (dB) via speakers in an enclosed room. Participants were comfortably seated and instructed to watch a muted movie to distract their attention from the sounds.

### ERP analyses of MMN

The EEG acquisition and pre-processing followed a standard procedure (see Supplementary Information). Pre-processed trials with voltages exceeding ±100 μV were regarded as contaminated by artifacts and automatically excluded by our customized script in MATLAB. As the number of standard trials far exceeded the number of deviant trials, a subset of standard trials was randomly selected to balance the number of standard and deviant stimulus conditions. Pre-processed trial epochs were averaged as a function of stimulus condition. Three difference waves (frequency, duration, and frequency-duration) were calculated by subtracting the average standard response from the average response to the corresponding deviant. Difference waves were separately averaged for each group at each placebo/drug condition to achieve grand average ERP waveforms. MMN was defined as the negative peak [100, 200] ms post-stimulus onset [68, 69]. The grand average latencies of the frequency MMN and the frequency-duration MMN were in the [100, 200] ms window; the duration MMN was prolonged to [200, 300] ms. To improve analytic consistency, a time window for individual MMN ERP features including the peak amplitude and its corresponding latency was defined as [100, 300] ms regardless of stimulus conditions.

### ERP analyses of responses to standard and deviant tones

Neural responses to each stimulus condition were examined without subtraction. The ERP waveforms were averaged as a function of the stimulus condition and the drug administration for TD and ASD. We focused on two prominent ERP components in the grand average waveforms: (i) The P1 (P50) component—a positive peak within [50, 100] ms post-stimulus onset; (ii) The N1 component following P1 as a negative valley in the range [100, 200] ms. In response to the duration deviant, there was another negative valley (N2) in the range of [200, 300] ms, with an amplitude similar to N1 evoked by the frequency deviant and the combined deviant, larger than the N1 component of the duration deviant. The temporal range of this N2 component was aligned with the duration MMN. Thus, the window for individual measurement of the negative component of the duration deviant was set as [200, 300] ms (not [100, 200] ms for other stimuli), to ensure the same neural component contributing to the MMN was measured across stimulus conditions. The amplitudes of the P1 and N1 were extracted for each participant for statistical analysis.

### Time-frequency analyses of responses to standard and deviant tones

ERSP analysis assessed dynamic changes in spectral bands after stimulus onset. The single-trial epochs used in the ERP analyses were inputted into the ERSP function ‘newtimef’ implemented with EEGLAB. The time limits of the trial epochs were [−100, 500] ms referenced to the stimulus onset, with sampling rate 250 Hz. The frequency limits were set as [4, 25] Hz with 0.5 Hz frequency resolution. Baseline spectra were calculated from the pre-stimulus interval [−50, 0] ms. Each epoch was divided into overlapping Hanning windows—range 128 ms (32 samples), step-size 4 ms. Spectra of each window were normalized with reference to the baseline and assigned to the center point of the window. Time limits of the output ERSP were [−36, 436] ms referenced to the stimulus onset. For each stimulus condition, the normalized transforms of single-trial epochs were then averaged and recorded as log values in a time-by-frequency ERSP matrix. Grand average ERSP under each stimulus condition was then calculated by averaging the time-by-frequency ERSP matrix across participants grouped by the ASD/TD and placebo/drug conditions.

Grand average ERSP outcomes of participants grouped by the placebo/drug condition were calculated by averaging ERSP matrices in each group. The main event-related perturbations at the grand average level occurred within the theta and the alpha band [4, 12] Hz. Therefore, for each participant visit, ERSP values in the [4, 12] Hz band were summed up to obtain the evoked power waveform. The mean of the evoked power waveform within the specified time window was extracted as a single-point measurement of event-related spectral dynamics. The measurement windows used for the four stimulus conditions were the same as the N1 settings in the ERP analyses—([200, 300] ms for the duration deviant, [100, 200] ms for others). The ERP and ERSP procedures were applied for post hoc comparisons between responses to pre-deviant standards and post-deviant standards (see Supplementary Information).

### Statistical analyses

The Shapiro-Wilk test was used to verify the normality of the ERP and ERSP responses of the TD and ASD groups under different experimental conditions. Extreme points (two for TD, three for ASD) located outside two standard deviations were excluded prior to the analysis. A paired-sample *t* test was performed for the TD and the ASD group separately for pair-wise comparisons between each pair of stimulus conditions at placebo/drug administrations. Under each stimulus condition, an independent-sample *t* test was used to assess group differences in ERP and ERSP responses at placebo/drug administrations. Paired-samples *t* test was also used for post hoc within-group comparisons between ERSP responses at placebo and 30 mg. Multiple comparisons were corrected using the Benjamini–Hochberg method [70]. Correlation analyses were performed between the AQ scores (total and Q5) and the placebo-30 mg shift in ERSP responses to pre-deviant standard tones. Reported *r* values are Pearson’s linear correlation coefficients; associated *p* values were computed using a Student’s t distribution for a transformation of the correlation.

Under each stimulus condition, a linear mixed-effect model (LMM) was built with drug dose and group as fixed-effect variables and subject as random-effect variable. This provided a measure of differential modulations by arbaclofen in TD and ASD as a group-drug interaction. When a significant group-drug interaction was observed, the LMM model shrank to a simple linear model with drug dose as the fixed-effect variable and subject as the random-effect variable, separately applied to TD and ASD. Finally, age was added as a fixed variable to the LMM model and the analysis was repeated to control for the age effect on the main outcomes.

## Results

### Clinical cohort

Thirty-eight TD (22 males, 16 females) and 28 ASD (20 males, 8 females) were included; 138 study visits were completed: 50 placebo (P) (26 TD, 24 ASD), 50 low-dose (L) (30 TD, 20 ASD), 38 high-dose (H) (19 TD, 19 ASD). Please see Table 1 for demographics. The null hypothesis held for all of the Shapiro-Wilk normality tests of the ERP and ERSP responses of the TD and ASD groups under different experimental conditions.

### MMN was comparable in ASD and TD; arbaclofen had a minimal impact on the MMN in the TD group only

**Fig. 1:**
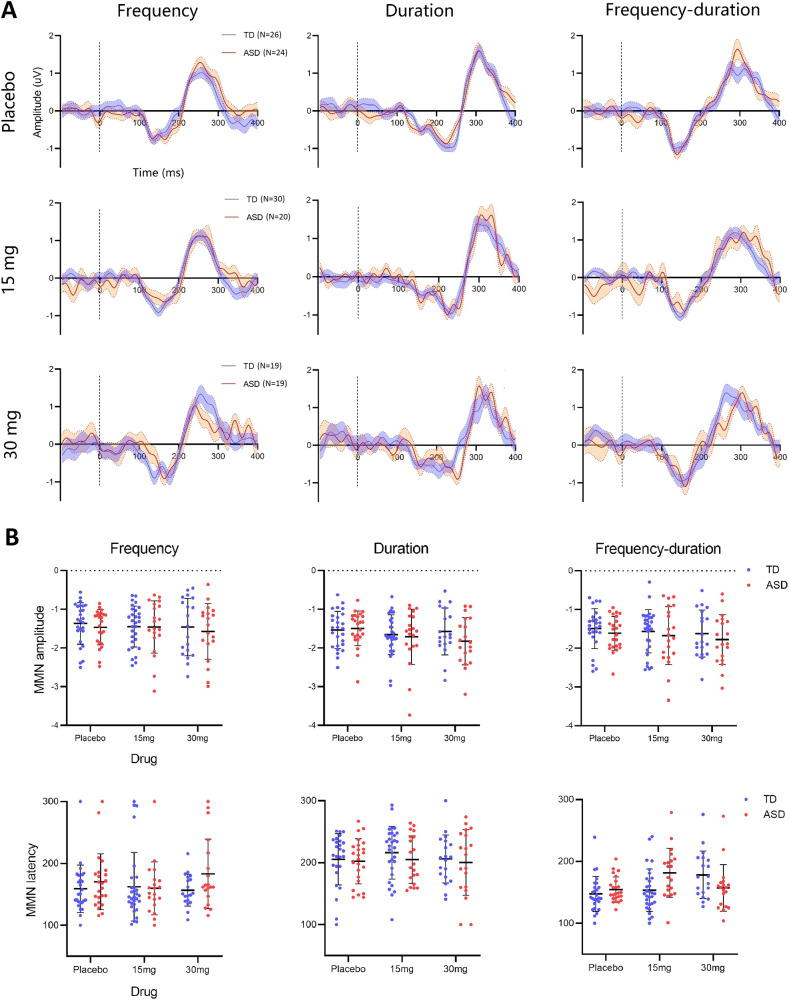
ERP waveforms and individual features of MMN. **A** The MMN grand average waveforms of the TD group (blue) and ASD group (red) as functions of the stimulus conditions (column) and drug administrations (row). Data epoch was drawn in the interval [−100, 400] ms referenced to the stimulus onset at 0 ms. Dashed lines indicate the stimulus onsets. **B** Individual scatter plots of MMN amplitudes (uV) and latencies (ms). *N* number of participants. Error bar shows the standard deviants (SD).

As shown in Fig. 1A, the MMN appeared as a negative valley in the grand average waveforms [100, 250] ms post stimulus onset and persisted across stimulus conditions and drug administrations in both groups. Individual-level ERP features (MMN amplitudes and corresponding latencies) are shown in Fig. 1B. There were neither significant group differences nor any group-drug interactions in the MMN amplitudes or latencies in any of the three deviant stimulus conditions (see Supplementary Information).

### Repetition suppression in ERP responses occurred in both TD and ASD; but suppression of P1 was significantly less in ASD

**Fig. 2:**
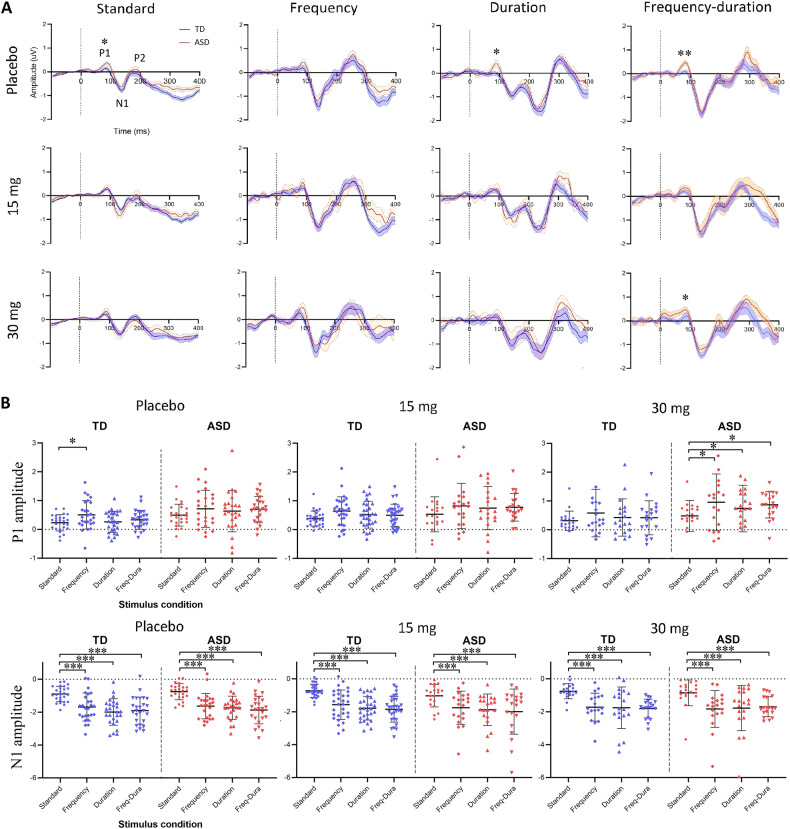
ERP waveforms and individual features of responses to standard tones and deviants. **A** The grand average waveform for the TD (blue) and ASD (red) group of ERP responses to standard tones and the three deviants (frequency, duration, and combined) without subtraction at the three-drug administrations (row). Dashed lines indicate the stimulus onsets. Components where a significant group difference was observed were marked with asterisks. **B** Individual scatter plots of the P1 and N1 amplitudes (µV) for the TD (blue) and ASD (red) group as a function of the stimulus condition at placebo/drug administration. The comparisons between stimulus conditions were achieved by paired-samples *t* test and corrected using the Benjamini–Hochberg method. *, the difference is statistically significant with corrected *p* < 0.05; **, the difference is statistically significant with corrected *p* < 0.01; ***, the difference is statistically significant with corrected *p* < 0.001. Error bars show SD.

Standalone grand average ERP waveforms of responses to the standard tones and the three deviants (frequency, duration, and frequency-duration) without subtraction are shown in Fig. 2A. Three prominent ERP components were observed in both the TD group and the ASD group: (i) an early P1 component appeared as a positive peak within the range [50, 100] ms post stimulus onset; (ii) a negative N1 components located within [100, 200] ms; (iii) a late P2 component followed N1 as a positive peak. The P2 component mostly contributed to the P3a after the MMN (Fig. 1A) and was therefore not further examined. For the duration deviant, there were two negative valleys located within [100, 300] ms. The latter was used as it aligned with the temporal range of the duration MMN (Fig. 1A).

At placebo, the ASD group had significantly higher P1 amplitudes than TD in response to standard tones (*t*_(48)_ = 2.8, *p* = 0.01), duration deviants (*t*_(48)_ = 2.3, *p* = 0.03) and combined deviants (*t*_(48)_ = 3.3, *p* = 0.004); the mean of ASD in response to frequency deviants was also higher than TD, though this difference was not significant (*t*_(48)_ = 1.3, *p* = 0.2). Individual scatter plots of P1 amplitudes are shown in Fig. 2B (top). At placebo, P1 amplitudes to repeated standard tones were significantly attenuated (suppressed) relative to those to frequency deviants in TD (*t*_(25)_ = 2.8, *p* = 0.03), but not in ASD. At 30 mg arbaclofen, P1 amplitudes to standards in ASD were significantly suppressed compared with those to the three deviants (frequency: *t*_(18)_ = 2.6, *p* = 0.04; duration: *t*_(18)_ = 2.7, *p* = 0.04; frequency-duration: *t*_(18)_ = 2.5, *p* = 0.04), while there was no difference between responses to standards and any deviants in TD. There was no difference in response between any pair of the three deviants. No drug effect or group-drug interaction was observed for TD or ASD under any condition. Statistics reported were corrected using the Benjamini–Hochberg method.

Individual scatter plots of the amplitudes of the N1 component are shown in Fig. 2B (bottom). At placebo and drug administrations, N1 amplitudes to standard tones were significantly suppressed relative to any of the three deviants in both the TD and ASD groups. Statistical results of N1 comparisons between each pair of stimulus conditions are shown in Table S1 in Supplementary Information. There was no difference in response between any pair of the three deviants. No group difference, drug effect or group-drug interaction was observed at any stimulus condition.

### Repetition suppression of spectral responses was weaker in ASD; arbaclofen shifted spectral responses to a more typical profile in ASD but disrupted spectral response in TD

#### Qualitative observations

**Fig. 3:**
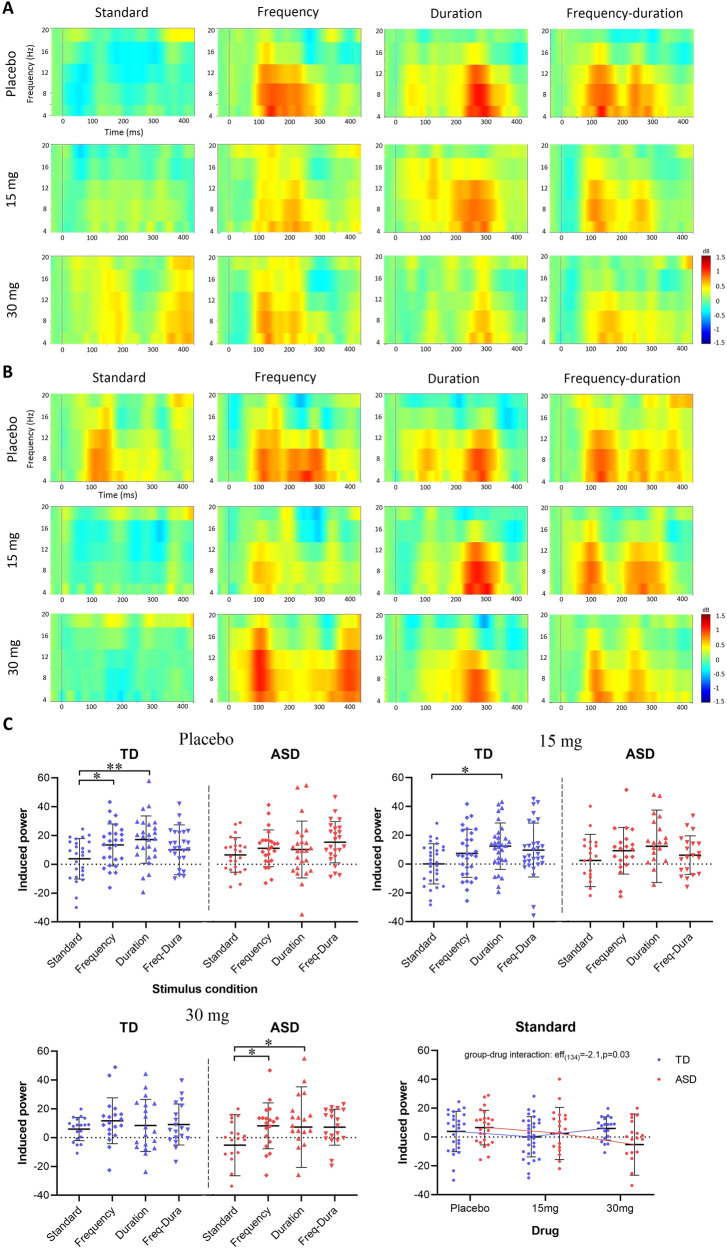
ERSP responses to standard tones and deviants. The grand average ERSP responses under different stimulus and drug conditions are presented for the TD group (**A**) and the ASD group (**B**). The *x* axis shows the time, and the *y* axis indicates spectral permutations within the [4, 20] Hz band. Dashed lines indicate the stimulus onsets. **C** Scatter plots of ERSP responses in [4, 12] Hz are presented for the TD (blue) and ASD (red) group at placebo (top left), 15 mg (top right), and 30 mg (bottom left). The *x* axis indicates the stimulus conditions. ERSP responses to repeated standards are separately displayed (bottom right) to show the group-drug interaction measured by LMM. The comparisons between stimulus conditions were achieved by paired-sample *t* test and corrected using the Benjamini–Hochberg method. *, the difference is statistically significant with corrected *p* < 0.05; **, the difference is statistically significant with corrected *p* < 0.01. Error bars show SD.

The ERSPs under different stimulus and drug conditions are presented in Fig. 3A, B. At placebo, repeated standard tones induced perturbations in the theta and alpha band [4, 12] Hz in ASD but spectral responses to repeated standards were clearly suppressed in TD. Low-dose (15 mg) and high-dose (30 mg) arbaclofen caused suppression of the standard-induced changes in ASD but reduced suppression in TD. The three deviants induced prominent spectral perturbations in both TD and ASD regardless of drug administration.

#### Quantitative observations

Scatter plots of the ERSP responses in the [4, 12] Hz band are shown in Fig. 3C. At placebo, spectral responses to repeated standard tones in TD were significantly suppressed compared with those to the frequency deviants (*t*_(25)_ = 2.2, *p* = 0.03) and the duration deviants (*t*_(25)_ = 3.7, *p* = 0.005), while no suppression was observed in ASD. At 15 mg, the suppression between standards and the duration deviants remained significant in TD (*t*_(29)_ = 3.7, *p* = 0.03). At 30 mg, spectral responses to repeated standard tones in ASD were significantly suppressed compared with those to the frequency deviants (*t*_(18)_ = 2.6, *p* = 0.03) and the duration deviants (*t*_(18)_ = 2.7, *p* = 0.03), while the typical suppression was disrupted in TD. There was no difference between any pair of the three deviants. The LMM confirmed a significant group-drug interaction (eff_(134)_ = −2.1, *p* = 0.03) in responses to standard tones. This was explained by a significant drug effect in spectral response to standards in ASD (eff_(61)_ = −2.3, *p* = 0.02) but not in TD (eff_(73)_ = 0.4, *p* = 0.7). No group difference, drug effect, or interaction was observed in responses to any of the three deviants. Statistics reported were corrected using the Benjamini–Hochberg method.

**Comparisons between pre- and post-deviant standards:**

1. **Repetition suppression to pre-deviant standards was significantly weaker in ASD and was rescued by arbaclofen**.
2. **Repetition suppression to pre-deviant standards was stronger in TD and disrupted by arbaclofen**.

**Fig. 4:**
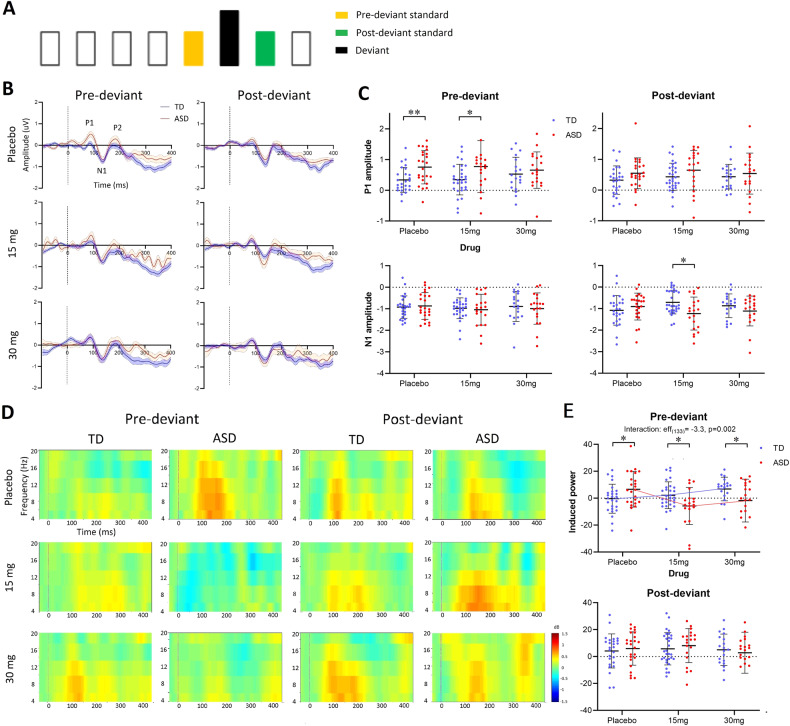
ERP and ERSP responses to pre- and post-deviant standards. **A** Timeline schematic of an example stimulus delivery that the pre-deviant standard (yellow) and post-deviant standard (green) are adjacent to the deviant (black). **B** The grand average waveforms of ERP responses to pre-deviant and post-deviant standards at different drug administrations for TD (blue) and ASD (red). Dashed lines indicate the stimulus onsets. **C** Scatter plots of the P1 (top) and N1 (bottom) amplitudes. The group differences were generated using an independent-samples *t* test and corrected using the Benjamini–Hochberg method. **D** The grand average ERSP responses to pre-deviant and post-deviant standards. **E** Scatter plots of ERSP responses in [4, 12] Hz band. The LMM group-drug interaction in ERSP responses to pre-deviant standards is displayed with broken mean lines.

In a repeated sequence, responses to later stimuli are expected to be more suppressed than responses to early stimuli, such as those at the beginning or after a deviant [2]. The ‘pre-deviant standard’ was defined as the last sound in a four-in-a-row standard sequence before a deviant. The ‘post-deviant standard’ was the first after a deviant (Fig. 4A).

#### ERP

The ERP waveforms and individual scatter plots of ERP components (P1 and N1) are shown in Fig. 4B, C. In the case of P1 amplitudes, at placebo, individuals in the ASD group had significantly higher responses to pre-deviant standards relative to TD (*t*_(48)_ = 3.1, *p* = 0.009); at 15 mg, the group difference remained significant but less so (*t*_(48)_ = 2.2, *p* = 0.04); at 30 mg, there was no difference between the two groups (*t*_(36)_ = 0.7, *p* = 0.5). No group-drug interaction or drug effects were observed by the LMM. There were no group differences, drug effects, or group-drug interaction in P1 amplitudes to the post-deviant standards. In the case of N1 amplitudes, there were no group differences, drug effect, or group-drug interaction observed in N1 to pre-deviant standards. A significant group difference was observed in N1 to post-deviant standards at 15 mg administration (*t*_(48)_ = 3.1, *p* = 0.02), while no group differences were observed at placebo or 30 mg and there were no drug effects or group-drug interaction.

#### ERSP

The grand average ERSP responses to pre- and post-deviant standards are shown in Fig. 4D. The group difference in standard responses previously observed in the placebo condition (Fig. 3) appeared driven by differences in pre-deviant but not post-deviant responses. At individual-level (Fig. 4E), significant group differences were observed following placebo and drug administrations in responses to pre-deviant standards (at placebo, *t*_(48)_ = 2.1, *p* = 0.04; at 15 mg, *t*_(48)_ = −2.3, *p* = 0.04; at 30 mg, *t*_(48)_ = −2.1, *p* = 0.04) but not to post-deviant standards. LMM results confirmed a strong group-drug interaction in responses to pre-deviant standards (eff_(133)_ = −3.3, *p* = 0.002), but not in responses to post-deviant standards. Specifically, spectral responses to pre-deviant standards increased with drug dose in TD (eff_(73)_ = 2.4, *p* = 0.01; weaker suppression with increasing dose) while they decreased in ASD (eff_(60)_ = −2.3, *p* = 0.02; stronger suppression with increasing dose). Statistics reported were corrected using the Benjamini–Hochberg method.

Although the age range of the ASD and TD groups was similar (20–51 years and 19–53 years respectively, see Table 1), the mean age of the ASD group (34.8 years) was higher than that of the TD group (28.6 years) (*t*_(66)_ = 2.7, *p* = 0.01). However, the LMM results with age controlled as a fixed variable confirmed there was no age effect observed in our main outcomes, such as the amplitude of the P1 component (eff_(132)_ = 0.3, *p* = 0.7) and the ERSP responses (eff_(132)_ = 0.18, *p* = 0.8) to pre-deviant standard tones. Thus, age difference was unlikely to explain any group differences reported.

### Individual sensitivity to GABA_B_ activation and relationship with wider autistic symptomatology

We defined a GABA_B_ ‘sensitivity index’ for each individual as the difference in spectral responses to pre-deviant standards at placebo minus those at 30 mg arbaclofen.

**Fig. 5:**
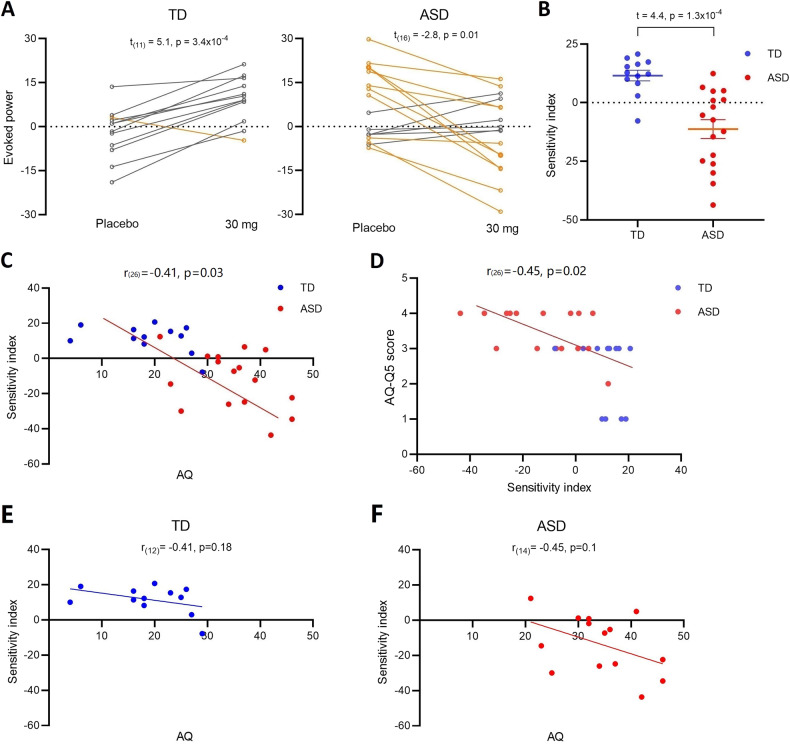
Individual sensitivity index and relationship with symptomatology. **A** The transition graphs show the shift effect between placebo and 30 mg arbaclofen on TD and ASD. Yellow lines indicate a decrease in evoked power following arbaclofen (the majority of the ASD group); gray lines indicate an increase in evoked power following arbaclofen (the majority of the TD group). Only one individual in the TD group (yellow) had a decrease in evoked power in response to arbaclofen (behaving more like the ASD group); that person also had the highest AQ score in the TD group. **B** The scatter plots of extracted sensitivity indexes. **C** The partial correlation between the sensitivity index and total AQ scores is significant after controlling for group. The point at which the direction of the sensitivity index changed (i.e., crosses the *x* axis) approximates the AQ ‘cut-off’ for ASD [60]. **D** The partial correlation between the sensitivity index and sound sensitivity captured by AQ question 5 score is significant after controlling for group. However, the within-group correlation did not reach significance in the TD (**E**) or ASD group (**F**) when examined separately.

The placebo-30 mg transitions in the two groups are shown in Fig. 5A. Eleven out of 12 (92%) TD participants showed an increased effect of drug—a significant within-group placebo-30 mg difference (*t*_(11)_ = 5.1, *p* = 3.4 × 10^–4^). In contrast, 11 out of 17 ASD (65%) showed a decreased effect of drug, generating a significant within-group placebo-30 mg difference (*t*_(16)_ = −2.8, *p* = 0.01). The group difference in the sensitivity index was also significant (*t*_(27)_ = 4.4, *p* = 1.3 × 10^–4^; Fig. 5B). There was a significant partial correlation between GABA_B_ response sensitivity and total scores on the AQ across the TD and ASD groups after controlling for group (*r*_(26)_ = −0.41, *p* = 0.03; Fig. 5C); and between response to AQ question 5: “I often notice small sounds when others do not” (*r*_(26)_ = −0.45, *p* = 0.02; Fig. 5D). However, as treating both groups as one sample can confound interpretation, we also examined within-group correlation with AQ. The correlation did not reach statistical significance in either ASD or TD, potentially due to the loss of power (TD: *r*_(12)_ = −0.41, *p* = 0.18; ASD: *r*_(14)_ = −0.45, *p* = 0.11).

To explore post hoc whether our data informed a possible stratification approach [22], we defined a stratification ‘threshold’ as the mean of baseline ERSP responses to pre-deviant standards in ASD. We first confirmed almost all TD participants who completed both placebo and high-dose visits were below this threshold (11 out of 12; 92%). Next, we used the stratification threshold to evenly divide the ASD group (17 in total) into two subgroups with 8 above (‘hyper’ group) and 9 below (‘hypo’ group) the threshold. Arbaclofen shifted the baseline ERSP responses down in all individuals from the ‘hyper’ group; however, it showed a heterogeneous effect in the ‘hypo’ group: baseline responses were shifted up in two-thirds of autistic participants and down in the remainder. A significant group-drug interaction was confirmed by LMM (eff = 2.4, *p* = 0.02).

## Discussion

Differences in sensory processing are core to ASD, and have been postulated to arise from alterations in excitation–inhibition balance [71], especially GABAergic dysfunction [72]. To directly test the hypothesis, that in humans, differential neural responses to auditory stimuli in ASD are underpinned by GABA, we used an auditory oddball paradigm. We confirmed our two main hypotheses: (i) Atypical repetition suppression in ASD relative to TD; (ii) Differential modulation of repetition suppression by the GABA system in ASD and TD. Specifically, we report weaker suppression in both temporal ERP and spectral ERSP responses to repetitive standard tones in ASD relative to TD at placebo (baseline), consistent with prior ERP work [2].

We then focused on the pre-deviant standards expected to be most affected by repetition suppression. We demonstrated, for the first time, that activating GABA_B_ receptors through a single oral dose of arbaclofen reversed atypical auditory processing in ASD and disrupted typical responses in TD. This is direct evidence of GABAergic differences in auditory neurophysiology in ASD.

We further moved beyond group-level approaches to capture the extent to which an individual responded to modulation of the GABA system with arbaclofen as its sensitivity index. This index strongly correlated with that individual’s ASD symptomology measured by the total AQ score. Moreover, a single AQ question capturing subjective sensitivity to sounds (perception), is also strongly correlated with GABAergic response. Thus, more autistic characteristics, including auditory perceptual features, were associated with greater establishment of repetition suppression by arbaclofen. The correlation analyses were under-taken to understand if the extent of GABA function differences at the sensory processing level are related to the extent of autistic features. We interpret the correlation result as indicating that the direction and extent of arbaclofen-elicited (i.e. GABA-dependent) shift in sensory processing is associated with the extent of an individual’s autistic features. Indeed, the transition between a weakening and strengthening suppression in response to arbaclofen was observed at AQ score 26—the ‘cut-off’ for ASD. In other words, GABA_B_ receptor activation has diametrically opposite effects on auditory processing in people who score above and below a phenotypic ‘cut-off’ for ASD. Importantly, our work indicates that auditory processing profiles are not ‘fixed’ in either ASD or TD; they can be modulated, even in adults.

### Adapting to repeated and novel stimuli

Our results indicate that in ASD there is a relative failure to dampen the response to repetitive and predictable information (weaker repetition suppression), while preserving the response to true deviants (normal deviance detection). These results can sit comfortably within a predictive coding [73] or feedforward adaptive filtration framework [34], as repetition suppression is manifest in either case.

#### Event-related MMN

There was no group difference at baseline and only a minimal effect of arbaclofen in either group on the ERP-measured MMN. This is consistent with previous negative results in oddball studies of ASD because the ERP used to calculate the MMN is largely determined by deviance detection rather than response suppression [41, 42], but the picture is complicated. This again can be explained within either predictive coding or feedforward adaptive filtering frameworks. The former postulates that deviance detection captured by the MMN relies on the formulation of a short-term trace of previous regularity and is memory-based, which may not need to be GABA-dependent [74, 75]. Given the considerable heterogeneity in memory-related phenotypes in ASD [76, 77], we might expect minimal group differences and/or response to GABAergic challenge on this metric. Therefore, we might expect minimal group differences and/or responses to GABAergic challenge on this metric. The latter theory only requires that synapses that are modified in response to the repeated stimulus are distinct from those that process the novel stimulus. It allows for event-related MMN to continue in both groups and be relatively unchanged by arbaclofen.

#### Repetition suppression

Preclinical repetition suppression (SSA) has been clearly demonstrated modulated by the GABA system [46, 47]. Our findings show that the repetition suppression (but not deviance detection) in our human participants was GABA-dependent, which is consistent with the preclinical literature. We further demonstrated that the repetition suppression was disrupted in autistic adults and arbaclofen ameliorated repetition suppression anomalies, with the most prominent group difference and drug modulation effect observed in responses to repeated standard stimuli at [50, 100] ms after stimulus onset. This time window of significance mainly corresponds to the P1 component and the transfer from the P1 to N1. Surface P1 receives bilateral contributions from the primary auditory cortex [78], and is assumed to reflect automatic ‘bottom-up’ processing of auditory inputs, such as feature extraction [79] and stream separation [80]. Thus, our findings may indicate a disruption in the function of GABA pathways underpinning the typical ‘bottom-up’ processing of auditory inputs in individuals with ASD.

### Heterogeneous neurophysiological responses to arbaclofen—clinical relevance

Activating GABA receptors through arbaclofen has been suggested as a potential therapeutic strategy for ASD [22]. Arbaclofen is the active right enantiomer of baclofen with >100 fold specificity for GABA_B_ receptor compared to the ‘S’ enantiomer and approximately five times greater specificity than racemic baclofen [81]. Importantly, it has been shown to be safe and well-tolerated in individuals with ASD [60]. The most common adverse events are agitation and irritability, which are much less than baclofen and can be typically resolved without dose changes [60]. Clinical Trials of arbaclofen did show some promise in subgroups, but failed to change the primary outcome measure overall [22]. Our results also show heterogeneity in the neurophysiological response to GABA_B_ agonism in ASD. The reasons for heterogeneous responses to arbaclofen are likely to be complex. The metabotropic GABA_B_ receptors provide tonic inhibition and regulate cellular excitability through both pre- and postsynaptic mechanisms [82, 83]. They also have crosstalk with the glutamate system, as well as GABA_A_ receptors. Furthermore, they may impact overall GABA production and breakdown, and likely exert broad downstream cellular effects including both inhibition and disinhibition [84]. Thus, boosting GABAergic function through arbaclofen may affect a range of mechanisms that differentially modulate the excitatory and inhibitory targets in individuals with and without ASD.

Our findings do not speak to the clinical efficacy of arbaclofen. Although clinical Trials of arbaclofen did show some promise in subgroups, they failed to change the primary outcome measure overall [22]. In conventional ASD trials, primary outcome measures generally rely upon measures of complex behaviors, for example, the Aberrant Behavior Checklist—Irritability, the Social Responsiveness Scale, and the Vineland Adaptive Behavior Scale [22, 60]. Such behaviors are shaped by complex gene-environment interactions throughout life. We activated the function of GABA pathways by arbaclofen in individuals with and without ASD and found that arbaclofen modified a neurobiological process (auditory repetition suppression) in ASD without examining its efficacy on complex behaviors. The path forward, we would suggest, is that understanding an individual’s GABA profile might help us establish if that individual has a GABA difference to target in a future study of clinical effectiveness of GABA-acting drugs such as arbaclofen. The hope is that this will support future mechanism-informed trials of efficacy.

### Cellular and developmental bases of repetition suppression differences

Though we cannot establish what cellular differences in ASD explain the weak repetition suppression, there are converging observations from preclinical studies. First, it has been demonstrated that GABAergic somatostatin-positive interneurons (SOMs) selectively suppress responses to repetitive standards but not deviants in the primary auditory cortex in mice [85]. Second, others have found that silencing SOMs in rodents leads to the loss of rodent visual MMN [86]. Third, SOMs show late input/output facilitation in the MMN timescale [87] and have a slow firing rate which is well suited to serve as substrates of the slow theta, alpha, and beta rhythms supporting neuro-oscillatory responses to repeated stimuli [55] and the activity of SOMs is associated with cortical oscillations within these frequency bands [88, 89]. Thus, a candidate mechanism for weaker repetition suppression in ASD and differential GABAergic function may be altered interaction of SOMs with the pyramidal neurons in local circuits along hierarchical auditory pathways.

Preclinical examination of SSA suggests that postsynaptic GABA_B_ receptor activity is needed to reduce pyramidal response to repeated sounds, whereas presynaptic GABA_B_ receptor activity promotes responses to repeated sounds [47]. Speculatively, the postsynaptic GABA_B_ receptors response is dominant in the suppression in TD. Excessive GABA_B_ receptors activation with arbaclofen may shift the typical balance towards presynaptic GABA_B_ receptor activity and disrupt suppression. In ASD, since arbaclofen increases repetition suppression, this could indicate that postsynaptic GABA_B_ receptor mechanisms are altered at baseline; but further experimental work in animal models will be needed to test this concept.

### Origins of GABA differences in ASD

Our participants were adults, but ASD is a neurodevelopmental condition with origins in early life. Sensory circuits in which GABA has a key role mature in early postnatal periods and subsequent brain development ‘cascades’ through multiple sensitive periods as more and more complex cognitive and behavioral skills are acquired [90]. Indeed, atypical sensory processing has been flagged early in the development of infants who go on to receive a diagnosis of ASD [2, 91]. Thus, the atypical GABAergic auditory processing in ASD observed here is likely to reflect earlier alterations in neural circuit maturation. Consistent with this, others have reported altered auditory cortical reactivity in newborn infants at high risk of ASD [2, 62]. These GABAergic developmental perturbations are unlikely to be restricted to the auditory domain. We have documented tight links between the GABA system and altered tactile processing in ASD [63] and GABA-dependent differences in fundamental visual processing in ASD, which are also shifted to a more typical pattern with arbaclofen [64]. Prospective longitudinal studies will help map causal pathways from sensory abnormalities to later emerging symptoms in ASD.

### Limitations

This study has limitations. Though a considerable number of study visits had been completed, the total number of participants involved was relatively small, especially for those who completed both placebo and high-dose visits. This limited the power of the within-group correlation analysis between the P-H sensitivity index and AQ scores. Our participant cohort comprised solely of adults (given the ethical constraints of experimental pharmaco-challenge studies in children), thus our findings do not speak to when the autistic differences in auditory electrophysiological responses to GABA_B_ challenge rise during the development. Moreover, this study targeted high-functioning ASD with IQ > 70, therefore it cannot determine whether the observed differences apply to the broader autism spectrum. Future work will be needed and ideally should be larger-scale and multi-center to confirm that there is GABAergic modulation of auditory (or other sensory) features in ASD across age groups and clinical phenotypes. The hope is that this will support future mechanism-informed trials of efficacy at a steady state. It’s also worth exploring whether any effect of sex influences the GABA-dependent responses to sensory stimuli in individuals with ASD as sex differences in auditory processing are reported on different animal species, including humans [92].

### Conclusions

Our results suggest differential GABAergic regulation of auditory sensory processing in individuals with ASD; and show that GABA_B_ agonism shifts the autistic sensory profile towards a typical profile. If confirmed, they may provide a means to identify GABA-dependent sensory differences at the level of the individual and indicate that these are upstream of perceptual and more complex autistic features. GABA-dependent sensory alterations may therefore offer a mechanism-informed treatment target for some individuals with ASD, which could potentially have implications for broader function.

### Supplementary information

Supplementary Materials

## Data Availability

Data from this study are available on request.

## References

[1] American Psychiatric Association. Diagnostic and statistical manual of mental disorders. 5th edn., 2013.

[2] Kolesnik A, Begum Ali J, Gliga T, Guiraud J, Charman T, Johnson MH (2019). Increased cortical reactivity to repeated tones at 8 months in infants with later ASD. Transl Psychiatry.

[3] Tomchek SD, Dunn W (2007). Sensory processing in children with and without autism: a comparative study using the short sensory profile. Am J Occup Ther.

[4] Rosenhall U, Nordin V, Sandström M, Ahlsen G, Gillberg C (1999). Autism and hearing loss. J Autism Dev Disord.

[5] Kern JK, Trivedi MH, Garver CR, Grannemann BD, Andrews AA, Savla JS (2006). The pattern of sensory processing abnormalities in autism. Autism.

[6] Kuhl PK, Coffey‐Corina S, Padden D, Dawson G (2005). Links between social and linguistic processing of speech in preschool children with autism: behavioral and electrophysiological measures. Dev Sci.

[7] Ben-Sasson A, Hen L, Fluss R, Cermak SA, Engel-Yeger B, Gal E (2009). A meta-analysis of sensory modulation symptoms in individuals with autism spectrum disorders. J Autism Dev Disord.

[8] Robertson CE, Baron-Cohen S (2017). Sensory perception in autism. Nat Rev Neurosci.

[9] O’Connor K (2012). Auditory processing in autism spectrum disorder: a review. Neurosci Biobehav Rev.

[10] Baum SH, Stevenson RA, Wallace MT (2015). Behavioral, perceptual, and neural alterations in sensory and multisensory function in autism spectrum disorder. Prog Neurobiol.

[11] Rubenstein J, Merzenich MM (2003). Model of autism: increased ratio of excitation/inhibition in key neural systems. Genes Brain Behav.

[12] Levin AR, Nelson CA (2015). Inhibition-based biomarkers for autism spectrum disorder. Neurotherapeutics.

[13] Fatemi SH, Reutiman TJ, Folsom TD, Thuras PD (2009). GABA A receptor downregulation in brains of subjects with autism. J Autism Dev Disord.

[14] Han S, Tai C, Westenbroek RE, Frank HY, Cheah CS, Potter GB (2012). Autistic-like behaviour in Scn1a+/− mice and rescue by enhanced GABA-mediated neurotransmission. Nature.

[15] Chao H-T, Chen H, Samaco RC, Xue M, Chahrour M, Yoo J (2010). Dysfunction in GABA signalling mediates autism-like stereotypies and Rett syndrome phenotypes. Nature.

[16] Gaetz W, Bloy L, Wang D, Port RG, Blaskey L, Levy S (2014). GABA estimation in the brains of children on the autism spectrum: measurement precision and regional cortical variation. Neuroimage.

[17] Adhya D, Swarup V, Nagy R, Dutan L, Shum C, Valencia-Alarcón EP (2021). Atypical neurogenesis in induced pluripotent stem cells from autistic individuals. Biol Psychiatry.

[18] Paulsen B, Velasco S, Kedaigle AJ, Pigoni M, Quadrato G, Deo AJ (2022). Autism genes converge on asynchronous development of shared neuron classes. Nature.

[19] Robertson CE, Ratai E-M, Kanwisher N (2016). Reduced GABAergic action in the autistic brain. Curr Biol.

[20] Oblak AL, Gibbs TT, Blatt GJ (2010). Decreased GABAB receptors in the cingulate cortex and fusiform gyrus in autism. J Neurochem.

[21] Berry-Kravis EM, Hessl D, Rathmell B, Zarevics P, Cherubini M, Walton-Bowen K (2012). Effects of STX209 (arbaclofen) on neurobehavioral function in children and adults with fragile X syndrome: a randomized, controlled, phase 2 trial. Sci Transl Med.

[22] Veenstra-VanderWeele J, Cook EH, King BH, Zarevics P, Cherubini M, Walton-Bowen K (2017). Arbaclofen in children and adolescents with autism spectrum disorder: a randomized, controlled, phase 2 trial. Neuropsychopharmacology.

[23] Den Ouden HE, Friston KJ, Daw ND, McIntosh AR, Stephan KE (2009). A dual role for prediction error in associative learning. Cereb cortex.

[24] Escera C, Leung S, Grimm S (2014). Deviance detection based on regularity encoding along the auditory hierarchy: electrophysiological evidence in humans. Brain Topogr.

[25] Tivadar RI, Knight RT, Tzovara A. Automatic sensory predictions: a review of predictive mechanisms in the brain and their link to conscious processing. *Front. Hum. Neurosci.* 2021;15:702520. 10.3389/fnhum.2021.702520 PMC8416526 34489663

[26] Näätänen R, Gaillard AWK, Mäntysalo S (1978). Early selective-attention effect on evoked potential reinterpreted. Acta Psychol.

[27] Näätänen R, Paavilainen P, Rinne T, Alho K (2007). The mismatch negativity (MMN) in basic research of central auditory processing: a review. Clin Neurophysiol.

[28] Alho K (1995). Cerebral generators of mismatch negativity (MMN) and its magnetic counterpart (MMNm) elicited by sound changes. Ear Hear.

[29] Harms L, Michie PT, Näätänen R (2016). Criteria for determining whether mismatch responses exist in animal models: focus on rodents. Biol Psychol.

[30] Todd J, Harms L, Schall U, Michie PT (2013). Mismatch negativity: translating the potential. Front psychiatry.

[31] Ulanovsky N, Las L, Nelken I (2003). Processing of low-probability sounds by cortical neurons. Nat Neurosci.

[32] Garrido MI, Kilner JM, Stephan KE, Friston KJ (2009). The mismatch negativity: a review of underlying mechanisms. Clin Neurophysiol.

[33] Heilbron M, Chait M (2018). Great expectations: is there evidence for predictive coding in auditory cortex?. Neuroscience.

[34] Ramaswami M (2014). Network plasticity in adaptive filtering and behavioral habituation. Neuron.

[35] Jääskeläinen IP, Ahveninen J, Bonmassar G, Dale AM, Ilmoniemi RJ, Levänen S (2004). Human posterior auditory cortex gates novel sounds to consciousness. Proc Natl Acad Sci.

[36] Parras GG, Nieto-Diego J, Carbajal GV, Valdés-Baizabal C, Escera C, Malmierca MS (2017). Neurons along the auditory pathway exhibit a hierarchical organization of prediction error. Nat Commun.

[37] Ciarrusta J, Dimitrova R, Batalle D, O’Muircheartaigh J, Cordero-Grande L, Price A (2020). Emerging functional connectivity differences in newborn infants vulnerable to autism spectrum disorders. Transl Psychiatry.

[38] Ciarrusta J, O’Muircheartaigh J, Dimitrova R, Batalle D, Cordero-Grande L, Price A (2019). Social brain functional maturation in newborn infants with and without a family history of autism spectrum disorder. JAMA Netw open.

[39] Vlaskamp C, Oranje B, Madsen GF, Møllegaard Jepsen JR, Durston S, Cantio C (2017). Auditory processing in autism spectrum disorder: Mismatch negativity deficits. Autism Res.

[40] Dunn MA, Gomes H, Gravel J (2008). Mismatch negativity in children with autism and typical development. J Autism Dev Disord.

[41] Knight EJ, Oakes L, Hyman SL, Freedman EG, Foxe JJ (2020). Individuals with autism have no detectable deficit in neural markers of prediction error when presented with auditory rhythms of varied temporal complexity. Autism Res.

[42] Schwartz S, Shinn-Cunningham B, Tager-Flusberg H (2018). Meta-analysis and systematic review of the literature characterizing auditory mismatch negativity in individuals with autism. Neurosci Biobehav Rev.

[43] Carbajal GV, Malmierca MS (2018). The neuronal basis of predictive coding along the auditory pathway: from the subcortical roots to cortical deviance detection. Trends Hear.

[44] Alexander WH, Brown JW (2014). A general role for medial prefrontal cortex in event prediction. Front Comput Neurosci.

[45] Casado-Román L, Carbajal GV, Pérez-González D, Malmierca MS (2020). Prediction error signaling explains neuronal mismatch responses in the medial prefrontal cortex. PLoS Biol.

[46] Duque D, Malmierca MS, Caspary DM (2014). Modulation of stimulus‐specific adaptation by GABAA receptor activation or blockade in the medial geniculate body of the anaesthetized rat. J Physiol.

[47] Ayala YA, Malmierca MS (2018). The effect of inhibition on stimulus-specific adaptation in the inferior colliculus. Brain Struct Funct.

[48] Morgan CJ, Mofeez A, Brandner B, Bromley L, Curran HV (2004). Acute effects of ketamine on memory systems and psychotic symptoms in healthy volunteers. Neuropsychopharmacology.

[49] Newcomer JW, Farber NB, Jevtovic-Todorovic V, Selke G, Melson AK, Hershey T (1999). Ketamine-induced NMDA receptor hypofunction as a model of memory impairment and psychosis. Neuropsychopharmacology.

[50] Morgan CJ, Curran HV (2006). Acute and chronic effects of ketamine upon human memory: a review. Psychopharmacology.

[51] Makeig S (1993). Auditory event-related dynamics of the EEG spectrum and effects of exposure to tones. Electroencephalogr Clin Neurophysiol.

[52] Javitt DC, Lee M, Kantrowitz JT, Martinez A (2018). Mismatch negativity as a biomarker of theta band oscillatory dysfunction in schizophrenia. Schizophr Res.

[53] Ko D, Kwon S, Lee G-T, Im CH, Kim KH, Jung K-Y (2012). Theta oscillation related to the auditory discrimination process in mismatch negativity: oddball versus control paradigm. J Clin Neurol.

[54] Lee M, Sehatpour P, Hoptman MJ, Lakatos P, Dias EC, Kantrowitz JT (2017). Neural mechanisms of mismatch negativity dysfunction in schizophrenia. Mol Psychiatry.

[55] Womelsdorf T, Valiante TA, Sahin NT, Miller KJ, Tiesinga P (2014). Dynamic circuit motifs underlying rhythmic gain control, gating and integration. Nat Neurosci.

[56] Molyneaux BJ, Hasselmo ME (2002). GABAB presynaptic inhibition has an in vivo time constant sufficiently rapid to allow modulation at theta frequency. J Neurophysiol.

[57] Kessler K, Seymour RA, Rippon G (2016). Brain oscillations and connectivity in autism spectrum disorders (ASD): new approaches to methodology, measurement and modelling. Neurosci Biobehav Rev.

[58] Holiga Š, Hipp JF, Chatham CH, Garces P, Spooren W, D’Ardhuy XL (2019). Patients with autism spectrum disorders display reproducible functional connectivity alterations. Sci Transl Med.

[59] Langer N, Ho EJ, Alexander LM, Xu HY, Jozanovic RK, Henin S (2017). A resource for assessing information processing in the developing brain using EEG and eye tracking. Sci Data.

[60] Erickson CA, Veenstra-Vanderweele JM, Melmed RD, McCracken JT, Ginsberg LD, Sikich L (2014). STX209 (arbaclofen) for autism spectrum disorders: an 8-week open-label study. J Autism Dev Disord.

[61] Millin R, Kolodny T, Flevaris AV, Kale AM, Schallmo M-P, Gerdts J (2018). Reduced auditory cortical adaptation in autism spectrum disorder. ELife.

[62] Begum-Ali J, Kolesnik-Taylor A, Quiroz I, Mason L, Garg S, Green J (2021). Early differences in auditory processing relate to autism spectrum disorder traits in infants with neurofibromatosis type I. J Neurodev Disord.

[63] Puts NA, Wodka EL, Harris AD, Crocetti D, Tommerdahl M, Mostofsky SH (2017). Reduced GABA and altered somatosensory function in children with autism spectrum disorder. Autism Res.

[64] Huang Q, Pereira AC, Velthuis H, Wong NM, Ellis CL, Ponteduro FM (2022). GABAB receptor modulation of visual sensory processing in adults with and without autism spectrum disorder. Sci Transl Med.

[65] Sanchez-Ponce R, Wang L-Q, Lu W, Von Hehn J, Cherubini M, Rush R (2012). Metabolic and pharmacokinetic differentiation of STX209 and racemic baclofen in humans. Metabolites.

[66] Wechsler D. Wechsler Abbreviated Scale of Intelligence Second Edition (WASI-II) San Antonio. TX: Pearson, 2011.

[67] Baron-Cohen S, Wheelwright S, Skinner R, Martin J, Clubley E (2001). The autism-spectrum quotient (AQ): evidence from asperger syndrome/high-functioning autism, malesand females, scientists and mathematicians. J Autism Dev Disord.

[68] Näätänen R (2000). Mismatch negativity (MMN): perspectives for application. Int J Psychophysiol.

[69] Čeponienė R, Lepistö T, Shestakova A, Vanhala R, Alku P, Näätänen R (2003). Speech–sound-selective auditory impairment in children with autism: they can perceive but do not attend. Proc Natl Acad Sci.

[70] Benjamini Y, Hochberg Y (1995). Controlling the false discovery rate: a practical and powerful approach to multiple testing. J R Stat Soc: Ser B (Methodol).

[71] Foss-Feig JH, Adkinson BD, Ji JL, Yang G, Srihari VH, McPartland JC (2017). Searching for cross-diagnostic convergence: neural mechanisms governing excitation and inhibition balance in schizophrenia and autism spectrum disorders. Biol Psychiatry.

[72] Chen Q, Deister CA, Gao X, Guo B, Lynn-Jones T, Chen N (2020). Dysfunction of cortical GABAergic neurons leads to sensory hyper-reactivity in a Shank3 mouse model of ASD. Nat Neurosci.

[73] Sinha P, Kjelgaard MM, Gandhi TK, Tsourides K, Cardinaux AL, Pantazis D (2014). Autism as a disorder of prediction. Proc Natl Acad Sci.

[74] Umbricht D, Koller R, Vollenweider FX, Schmid L (2002). Mismatch negativity predicts psychotic experiences induced by NMDA receptor antagonist in healthy volunteers. Biol Psychiatry.

[75] Coffman BA, Haigh SM, Murphy TK, Salisbury DF (2017). Impairment in mismatch negativity but not repetition suppression in schizophrenia. Brain Topogr.

[76] Bennetto L, Pennington BF, Rogers SJ (1996). Intact and impaired memory functions in autism. Child Dev.

[77] Shalom DB (2003). Memory in autism: review and synthesis. Cortex.

[78] Godey B, Schwartz D, De Graaf J, Chauvel P, Liegeois-Chauvel C (2001). Neuromagnetic source localization of auditory evoked fields and intracerebral evoked potentials: a comparison of data in the same patients. Clin Neurophysiol.

[79] Näätänen R, Winkler I (1999). The concept of auditory stimulus representation in cognitive neuroscience. Psychol Bull.

[80] Gutschalk A, Micheyl C, Melcher JR, Rupp A, Scherg M, Oxenham AJ (2005). Neuromagnetic correlates of streaming in human auditory cortex. J Neurosci.

[81] Froestl W. Chemistry and pharmacology of GABAB receptor ligands. Adv Pharmacol. 2010;58:19–62. 10.1016/S1054-3589(10)58002-5 20655477

[82] Isaacson JS, Hille B (1997). GABAB-mediated presynaptic inhibition of excitatory transmission and synaptic vesicle dynamics in cultured hippocampal neurons. Neuron.

[83] Sohn JW, Lee D, Cho H, Lim W, Shin HS, Lee SH (2007). Receptor‐specific inhibition of GABAB‐activated K+ currents by muscarinic and metabotropic glutamate receptors in immature rat hippocampus. J Physiol.

[84] Connelly WM, Fyson SJ, Errington AC, McCafferty CP, Cope DW, Di Giovanni G (2013). GABAB receptors regulate extrasynaptic GABAA receptors. J Neurosci.

[85] Natan RG, Briguglio JJ, Mwilambwe-Tshilobo L, Jones SI, Aizenberg M, Goldberg EM, et al. Complementary control of sensory adaptation by two types of cortical interneurons. *Elife* 2015; **4**:e09868. 10.7554/eLife.09868 PMC4641469 26460542

[86] Hamm JP, Yuste R (2016). Somatostatin interneurons control a key component of mismatch negativity in mouse visual cortex. Cell Rep.

[87] Karnani MM, Agetsuma M, Yuste R (2014). A blanket of inhibition: functional inferences from dense inhibitory connectivity. Curr Opin Neurobiol.

[88] Chen G, Zhang Y, Li X, Zhao X, Ye Q, Lin Y (2017). Distinct inhibitory circuits orchestrate cortical beta and gamma band oscillations. Neuron.

[89] Hayden DJ, Montgomery DP, Cooke SF, Bear MF (2021). Visual recognition is heralded by shifts in local field potential oscillations and inhibitory networks in primary visual cortex. J Neurosci.

[90] Ciarrusta J, Dimitrova R, McAlonan G (2020). Early maturation of the social brain: how brain development provides a platform for the acquisition of social-cognitive competence. Prog Brain Res.

[91] He JL, Oeltzschner G, Mikkelsen M, Deronda A, Harris AD, Crocetti D (2021). Region-specific elevations of glutamate+ glutamine correlate with the sensory symptoms of autism spectrum disorders. Transl Psychiatry.

[92] Osório JMA, Rodríguez‐Herreros B, Richetin S, Junod V, Romascano D, Pittet V (2021). Sex differences in sensory processing in children with autism spectrum disorder. Autism Res.

