# Supplementary material for: Exploratory evidence for differences in GABAergic regulation of auditory processing in autism spectrum disorder
Source: Transl Psychiatry. 2023 Oct 18;13:320. doi: 10.1038/s41398-023-02619-8 (PMC10584846; doi:10.1038/s41398-023-02619-8)
Supplement: Supplementary file 1 — Supplementary Materials [file 41398_2023_2619_MOESM1_ESM.docx]

**SUPPLEMENTARY INFORMATION**

**Materials and Methods**

**Participants**

For the ASD group, 16 participants were recruited from our National Autism and ADHD service for Adults (NAASA) at the South London and Maudsley National Health Service (NHS) Foundation Trust; the service protocol includes an Autism Diagnostic Interview-Revised ^1^ or an Autism Diagnostic Observation Schedule ^2^ to inform the current symptom level. However, for inclusion in the ASD group of this study, we required a clinical diagnosis to be in place i.e. the final diagnostic decision was a clinical opinion made by an experienced psychiatrist or clinical psychologist from in a recognized ASD multidisciplinary clinical assessment setting in the UK. Those individuals recruited outside of our National Autism and ADHD Service for Adults (NAASA) at the South London and Maudsley NHS Foundation Trust were carefully screened by an experienced NAASA clinician for inclusion. The clinician had to be satisfied by the account of their diagnostic assessment through a recognized U.K. autism service (with documentary evidence where possible), in addition to their responses to our screening interview.

Participants with ASD with a known genetic cause, such as fragile X syndrome, neurofibromatosis type 1, or 22q11 deletion syndrome, were excluded from the study. Other inclusion criteria were as follows: ability to give informed consent, no comorbid psychiatric illness such as psychotic illness and major mood disorder, no history of seizures or diagnosis of epilepsy, and no physical illness, such as heart disease, high blood pressure, and renal insufficiency. In the month preceding participation, 13 participants (3 TD and 10 ASD) were taking regular medication with drugs such as sertraline, ibuprofen and citalopram, which did not affect glutamate or GABA directly. All other participants were medication-free.

**EEG acquisition and pre-processing**

Continuous scalp EEG signals were recorded via a 64-channel standard actiCAP (EASYCAP GmbH) with a sampling rate of 5 KHz and amplified by a BrainAmp amplifier (Brain Products GmbH). The default reference was FCz and electrode placements followed the international 10-20 system. Impedances between the scalp and electrodes were kept below 15 kꭥ.

Offline pre-processing was conducted using custom scripts that included functions from the EEGLAB ^3^ in MATLAB. Raw EEG data was first re-referenced to the mean of mastoids, down-sampled to 250 Hz and filtered between 0.1 and 30 Hz using zero-phase finite impulse response filters. Next, continuous EEG data was segmented for each stimulus trial in the interval [-100, 500] ms referenced to the stimulus onset at 0 ms. The Hyvärinen's fixed-point algorithm ^4^ for independent component analysis (ICA) was performed by calling the ‘fastICA’ function to visually detect potential artifacts. A single channel (FC1) over the front-central region was selected for further analysis because MMN was reported to reach maximum amplitude in this area ^5^. Trial epochs with voltages exceeded ±100 μV were regarded as contaminated by eye blinks or unexpected artifacts and excluded from analysis. For the frequency deviant, the averaged number of included trials per visit was 63.5±6.5 for TD and 58.8±12.1 for ASD (t=1.7, p=0.1); for the duration deviant, it was 75.7±7.6 for TD and 70.2±13.5 for ASD (t=1.8, p=0.09); for the combined deviant, it was 78.2±7.4 for TD and 72.6±15.3 for ASD (t=1.7, p=0.1).

**Post-hoc comparisons between responses to pre-deviant standards and post-deviant standards**

We applied both ERP and ERSP analyses to compare data epochs obtained from the pre-deviant standard trials and those from the post-deviant standard trials. The pre-deviant standard was defined to be the last sound in a four-in-a-row standard sequence before a deviant; the post-deviant standard was the first standard after a deviant with an interval of one second. The numbers of accepted trials were balanced between the pre- and post-deviant standards (96 trials per participant for each type). Individual ERSP was measured as the mean of a 50-ms interval around the average peak perturbation within the range of interest [50, 150] ms post stimulus onset. Other parameters used for the ERP and ERSP analyses were the same as the settings applied on standard responses in previous sections.

To measure how a single individual responded to arbaclofen, we defined a sensitivity index as the placebo-30 mg difference in spectral responses to pre-deviant standards. We calculated the index for the 29 participants (12 TD, 17 ASD) that completed both the placebo and 30 mg visits and ran an independent-sample t-test to confirm the group difference. Further, a correlation analysis between the sensitivity index and quantified ASD traits measured by the AQ (total score and score of response to question 5) was done to investigate the relationship with general ASD phenotype and sound perception, respectively.

**Results**

**MMN was comparable in ASD and TD; arbaclofen had a minimal impact on the MMN in the TD group only.**

We used linear mix-effect models (LMM) to perform a repeated measures analysis examining the effect of deviant characteristic, group difference and drug effect on the individual MMN amplitudes and latencies. The p-values reported were corrected for multiple comparisons using Benjamini-Hochberg method ^6^. Please see Materials and Methods for details of statistical analyses.

*MMN amplitud*es: Across the whole cohort, significant differences were observed between frequency-MMN and duration-MMN (t_(409)_ = -3.1, p = 0.004), frequency-MMN and combined-MMN (t_(409)_ = -2.8, p = 0.006), but not between duration-MMN and combined-MMN (t_(409)_ = 0.2, p = 0.8). There were no significant effects of group or drug nor any interactions in the MMN amplitudes in any of the three deviant stimulus conditions.

*MMN latencie*s: Across the whole cohort, significant differences were observed between duration-MMN and frequency-MMN (t_(409)_ = -8.5, p = 3.3x10^-16^), duration-MMN and combined-MMN (t_(409)_ = -9.4**,** p = 3.8x10^-19^), but not between frequency-MMN and combined-MMN (t_(409)_ = -0.9, p = 0.5). This pattern was expected as the duration deviant must necessarily alter MMN latencies. A significant drug effect on the latencies of the combined-MMN was found in TD (eff_(73)_ = 3**,** p = 0.02) but not in ASD (eff_(73)_ = 0.4**,** p = 0.7); but the group-drug interaction did not reach statistical significance. No group difference, drug effect or interaction was found for the frequency-MMN or duration-MMN.

**Repetition suppression in ERP responses occurred in both TD and ASD; but suppression of P1 was significantly less in ASD.**

At placebo and drug administrations, N1 amplitudes to standard tones were significantly suppressed relative to any of the three deviants in both the TD and ASD groups. Statistical results of N1 comparisons between each pair of stimulus conditions are shown in Table S1.

**Table S1** Comparisons of N1 amplitudes between stimulus conditions

| Stimulus pair | TD_P | TD_L | TD_H | ASD_P | ASD_L | ASD_H |
| --- | --- | --- | --- | --- | --- | --- |
| Std vs FD | t_(25)_=5.8  p=9x10^-6^ | t_(29)_=6.5  p=7.7x10^-7^ | t_(18)_=8  p=7.4x10^-7^ | t_(23)_=8.2  p=1.77x10^-7^ | t_(19)_=4.8  p=7.9x10^-4^ | t_(18)_=6.1  p=4.9x10^-5^ |
| Std vs DD | t_(25)_=8  p=1.3x10^-7^ | t_(29)_=8.2  p=1.5x10^-8^ | t_(18)_=3.8  p=0.003 | t_(23)_=7.1  p=6.8x10^-7^ | t_(19)_=4.4  p=9x10^-4^ | t_(18)_=4.3  p=8.7x10^-4^ |
| Std vs CD | t_(25)_=7.3  p=3.8x10^-7^ | t_(29)_=9.5  p=1.2x10^-9^ | t_(18)_=8.1  p=7.4x10^-7^ | t_(23)_=7.1  p=6.8x10^-7^ | t_(19)_=4.2  p=9x10^-4^ | t_(18)_=5.8  p=4.9x10^-5^ |
| FD vs DD | t_(25)_=1.6  p=0.17 | t_(29)_=1.3  p=0.25 | t_(18)_=0.13  p=0.9 | t_(23)_=0.57  p=0.6 | t_(19)_=0.52  p=0.64 | t_(18)_=0.17  p=0.8 |
| FD vs CD | t_(25)_=1.5  p=0.17 | t_(29)_=2.2  p=0.06 | t_(18)_=0.54  p=0.8 | t_(23)_=1.4  p=0.3 | t_(19)_=0.79  p=0.64 | t_(18)_=0.6  p=0.8 |
| DD vs CD | t_(25)_=0.45  p=0.6 | t_(29)_=0.37  p=0.7 | t_(18)_=0.18  p=0.9 | t_(23)_=0.71  p=0.57 | t_(19)_=0.47  p=0.6 | t_(18)_=0.27  p=0.8 |

Paired-samples t test was performed for the TD and the ASD group separately for pair-wise comparisons between each pair of stimulus conditions at placebo/drug administrations. Multiple comparison was corrected using the Benjamini-Hochberg method. TD_P: the TD group at placebo; TD_L: the TD group at 15 mg STX209; TD_H: the TD group at 30 mg STX209; ASD_P: the ASD group at placebo; ASD_L: the ASD group at 15 mg STX20; ASD_H: the ASD group at 30 mg STX209. Std: standard tone; FD: frequency deviant; DD: duration deviant; CD: combined frequency-duration deviant.

**Discussions**

**Sensory processing and wider autistic symptoms**

It was also beyond the scope of our study to examine how GABAergic modulation alters sensory *symptoms* (as opposed to sensory processing) in ASD, however, the strong correlation between the individual sensitivity index and AQ scores observed here supports the concept that the neuropathology underpinning atypical sensory suppression link to a wider phenotype.

Individuals with ASD often report that overwhelming sensory stimulation makes it difficult to interact with the environment ^7^. Differences in sensory processing have been identified across neurophysiology, perception and emotional responses to sensory inputs. Overwhelming sensory experiences have also been suggested to contribute to the anxiety which is extremely common in ASD, and predispose individuals with ASD to repetitive behaviors ^8^. Thus, sensory processing differences have tremendous impact in themselves and have knock-on effects on mental health and behavior. Our results suggest that the neurophysiology of auditory processing is linked with both sensitivity to sounds and wider phenotypes across our cohort as captured using the AQ. We show that the extent to which GABA-dependent neuro-oscillatory response (ERSP) to repeated auditory stimuli is atypical in ASD and related to an individual’s phenotype. This is potentially important because the neuro-oscillatory activity provides an essential platform for functional connectivity across brain networks ^9^. Therefore, our results raise the possibility that altered GABA-regulation of neuro-oscillatory responses to sensory stimuli has ‘knock-on’ effects across brain networks and the complex cognitive and behavioral functions they subserve. Investigations of such ‘fundamental’ sensory processes in ASD may therefore provide a more accessible route to modulating downstream difficulties experienced by autistic people.

**Arbaclofen side effects**

Known side effects of arbaclofen such as dizziness or nausea were reported by some participants occasionally throughout the study, but as expected, more so in the high-dose condition. For example, three participants with ASD reported fatigue and one TD participant reported for dizziness which were classified as moderate because they were more than ‘mild’. Mild side effects were very minimal, essentially any passing mention of side effects reflected a clear comment from the participant of a noticeable experience. This was not evaluated in terms of impact on function. These observed side effects could still potentially have limited acute dose studies by affecting the participants’ attentional resource allocation during the task. However, the auditory oddball paradigm used in this study was a passive paradigm with minimal demands on attention. The predictive response persists irrespective of attention during sleep ^10^ and anesthesia ^11^ and is detectable in early infancy ^12^, making it a promising tool for investigations of neurodevelopmental disorders ^13, 14^. We hope that this approach has a potential for generalization to individuals across age groups with immature or impaired cognitive abilities that are frequently excluded from drug development studies.

1. Lord C, Rutter M, Le Couteur A. Autism Diagnostic Interview-Revised: a revised version of a diagnostic interview for caregivers of individuals with possible pervasive developmental disorders. *Journal of autism and developmental disorders* 1994; **24**(5)**:** 659-685.

2. Lord C, Risi S, Lambrecht L, Cook EH, Leventhal BL, DiLavore PC *et al.* The Autism Diagnostic Observation Schedule—Generic: A standard measure of social and communication deficits associated with the spectrum of autism. *Journal of autism and developmental disorders* 2000; **30**(3)**:** 205-223.

3. Delorme A, Makeig S. EEGLAB: an open source toolbox for analysis of single-trial EEG dynamics including independent component analysis. *Journal of neuroscience methods* 2004; **134**(1)**:** 9-21.

4. Hyvärinen A, Oja E. A fast fixed-point algorithm for independent component analysis. *Neural computation* 1997; **9**(7)**:** 1483-1492.

5. Näätänen R, Paavilainen P, Rinne T, Alho K. The mismatch negativity (MMN) in basic research of central auditory processing: a review. *Clinical neurophysiology* 2007; **118**(12)**:** 2544-2590.

6. Benjamini Y, Hochberg Y. Controlling the false discovery rate: a practical and powerful approach to multiple testing. *Journal of the Royal statistical society: series B (Methodological)* 1995; **57**(1)**:** 289-300.

7. Tomchek SD, Dunn W. Sensory Processing in Children With and Without Autism: A Comparative Study Using the Short Sensory Profile. *The American Journal of Occupational Therapy* 2007; **61**(2)**:** 190-200.

8. Boyd BA, McBee M, Holtzclaw T, Baranek GT, Bodfish JW. Relationships among repetitive behaviors, sensory features, and executive functions in high functioning autism. *Research in autism spectrum disorders* 2009; **3**(4)**:** 959-966.

9. Pretzsch CM, Voinescu B, Mendez MA, Wichers R, Ajram L, Ivin G *et al.* The effect of cannabidiol (CBD) on low-frequency activity and functional connectivity in the brain of adults with and without autism spectrum disorder (ASD). *Journal of Psychopharmacology* 2019; **33**(9)**:** 1141-1148.

10. Strauss M, Sitt JD, King J-R, Elbaz M, Azizi L, Buiatti M *et al.* Disruption of hierarchical predictive coding during sleep. *Proceedings of the National Academy of Sciences* 2015; **112**(11)**:** E1353-E1362.

11. Quaedflieg CW, Münte S, Kalso E, Sambeth A. Effects of remifentanil on processing of auditory stimuli: A combined MEG/EEG study. *Journal of psychopharmacology* 2014; **28**(1)**:** 39-48.

12. Friederici AD, Friedrich M, Weber C. Neural manifestation of cognitive and precognitive mismatch detection in early infancy. *Neuroreport* 2002; **13**(10)**:** 1251-1254.

13. Näätänen R. Mismatch negativity: clinical research and possible applications. *International Journal of Psychophysiology* 2003; **48**(2)**:** 179-188.

14. Umbricht D, Krljes S. Mismatch negativity in schizophrenia: a meta-analysis. *Schizophrenia research* 2005; **76**(1)**:** 1-23.
